# A cluster randomized clinical trial of a stepped care intervention for depression in primary care (STEPCARE)- study protocol

**DOI:** 10.1186/s12888-015-0542-6

**Published:** 2015-07-07

**Authors:** Oye Gureje, Bibilola Damilola Oladeji, Ricardo Araya, Alan A. Montgomery

**Affiliations:** Department of Psychiatry, College of Medicine, University of Ibadan, Ibadan, Nigeria; Centre for Global Mental Health, London School of Hygiene and Tropical Medicine, London, UK; Nottingham Clinical Trials Unit, University of Nottingham, Queen’s Medical Centre, Nottingham, UK

**Keywords:** Depression, Primary health care, Stepped care

## Abstract

**Background:**

Depression constitutes a significant public health burden and is associated is with high level of individual suffering. Insufficient human and material resources impede the provision of adequate care for persons with the condition in low- and middle-income countries. It is commonly recognized that, to bridge this treatment gap, it is essential to integrate the treatment of depression into primary health care system.

**Methods/Design:**

STEPCARE is a two-arm parallel cluster randomized controlled trial to compare a stepped-care intervention package for depression in primary health care with care as usual in Nigeria. Randomization was conducted at the level of the participating primary health care clinics, while interventions are delivered to consenting individual participants who screen positive on the 9-item patient health questionnaire (PHQ-9 score ≥ 11) and fulfil the DSM-IV criteria for major depression. Intervention delivered by trained primary health care workers (PHCW) supported by general physicians and psychiatrists as needed is in 3 steps determined by response to treatment. Each step consists of psychological interventions (including psychoeducation, activity scheduling, social network reactivation and problem solving treatment) offered to all participants and, depending on severity and response, medication. Primary outcome, assessed at 12 months following recruitment into the trial, is recovery from depression as shown by a PHQ-9 score of less than 6. Secondary outcomes include changes in disability, quality of life and service utilization assessed at 6 and 12 months.

**Discussion:**

The stepped care model examines the effectiveness of an intervention package for depression in which the intensity of treatment is determined by the clinical need of the patients. This approach is designed to make the most efficient use of available resources.

**Trial registration:**

ISRCTN46754188 (ISRTCN registry at isrtcn.com; registered 23 September 2013)

## Background

It is estimated that depressive disorders will become the third most burdensome health problem in low income countries after HIV/AIDS and perinatal conditions by 2030 [1]. In Nigeria, the 12-month prevalence rates of depression in the general population is within a range of 1.5-7 % [2]. Studies in primary care show that depression is a common problem, occurring in up to 10-20 % of clinic attendees [3, 4]. Depression is strongly associated with poverty and social disadvantage [5] and is a risk factor for suicide, majority of which occurs in low and middle income countries (LMIC) [6]. Even though effective treatments for depression are available which, if provided, could alleviate the negative consequences of depression, a previous study in Nigeria showed that about four out of five persons with severe mental disorders, particularly depression, had received no treatment in the previous year and that, among those who did, only about 10 % received what could be considered as minimally adequate treatment [7, 8].

The Nigerian health system, similar to that in most of sub-Saharan Africa, is characterized by extreme resource constraints, both human and material. For example, there is only about one psychiatrist to a population of one million people and the few available specialists are inequitably concentrated in urban settings [9]. It is generally recognized that a way to minimize the consequences of this specialist manpower constraint is to integrate mental health (MH) into primary health care where services are mostly provided by non-physician primary health care workers (PHCWs). This strategy is much more likely to be viable and affordable because these resources already exist, are less expensive, and offer increased accessibility given that primary care clinics are closer to where people live. However this strategy is hampered by several factors: 1) inadequate training of the PHCWs, 2) lack of structured support and supervision for their work, and 3) competing duties [10]. The problem of lack of resources is further compounded by the inefficiency with which the limited available resources are used. For example, treatments lacking any evidence are often offered and specialist time and skill are not efficiently deployed. A new model to address the treatment gap for depression must therefore give prominence to a more efficient way of deploying existing resources to deliver effective interventions. Stepped-care models seek to maximize efficiency by deploying available resources strictly according to needs, offering greater resources to those with complex or severe problems [11].

There is now considerable evidence in support of stepped and collaborative care approaches to expanding mental health service [12, 13]. In this model, non-physician PHCWs deliver the bulk of essential mental health service under the supervision and support of nurses or physicians and occasionally of more highly trained mental health specialists, where these are available. This process, best described as task-sharing, facilitates the delivery of evidence-based health care even in the context of extreme shortage of specialists as seen in most LMIC. The World Health Organization (WHO) has produced a set of guidelines, the mental health gap action programme intervention guide (mhGAP-IG), that incorporates evidence-based interventions for a list of priority mental health conditions, including depression, to aid the management of these conditions in non-specialist settings [14]. It builds on the well-established knowledge that primary care providers can be trained to deliver both psychological and pharmacological interventions for several mental health conditions, while specialists offer necessary supervision and/or address more difficult or complex problems. The content of what is needed to scale up mental health services is therefore generally agreed upon. However, the mode of delivery of the intervention in diverse settings still requires empirical exploration in order to determine the best fit to local health systems.

There has been at least one systematic review of treatments for depression in LMIC [15]. This review shows that primary care providers can, with physician and specialist support, deliver effective interventions that include psychological as well as medication therapies. However, the studies demonstrating the feasibility of training primary care providers to deliver effective interventions for depression have, till date, been conducted in settings, which even though classified as LMIC, have better health resources than those existing in most of Sub-Sahara Africa. For example, a recent study from Goa, India, describes an effective stepped care approach in the treatment of common mental disorders in primary care settings [13]. All the facilities where the study was conducted had a physician as well as ready access to a psychiatrist. However, in Nigeria, as in most sub-Sahara African countries, in primary health care clinics (PHCCs) most services are provided by non-physician PHCWs with only sporadic in-house supervision from a physician. Specifically, in Nigeria, one general physician supervises an average of 8-15 primary care clinics. Also, in most of these countries, the few available psychiatrists are located in major cities, thus making access to them for support and supervision difficult if not impossible. The lack of adequate supervision and support for primary care providers is recognized as one of the major reasons for the failure of primary care to meet previous expectations in regard to provision of mental health services [10]. It is therefore important to design and study the effectiveness of an intervention package for depression that can be delivered almost exclusively by PHCWs with a feasible and affordable mode of obtaining supervision and support from physicians and specialists, wherever available.

## Methods and design

### Aim of the study

The main aim of this study is to test the effectiveness and cost-effectiveness of a stepped-care intervention program for depression in adults delivered mostly by non-physician PHCWs with medical and specialist supervision and support provided with the use of mobile phones compared to usual care in a randomized controlled trial.

### Design

This study is a two-arm parallel cluster randomized controlled trial comparing an intervention package for depression in primary care based on a version of the mhGAP-IG contextualized and adapted for the extant Nigerian health system to care as usual. The unit of randomization is the primary care clinic. As the intervention is designed to be delivered by clinic staff, the cluster randomized design was chosen in order to reduce the potential risk of contamination within clinics.

### Setting

The study is being conducted in Oyo State, Nigeria. The state has 33 local government areas (LGAs), from which eleven LGAs (five urban and six rural) were selected for the study. We included both urban and rural LGAs in order to capture the diversity of the socioeconomic profile of the country and because access to medical treatment differs substantially between these two types of setting, thus making the demonstration of the utility of our program potentially more generalizable. Our sampling frame included all the primary health care clinics (PHCCs) that have a full complement of primary care workers and provide a broad range of clinical services in the eleven LGAs (n = 97). Of these, 52 provided only maternal and child health care and were excluded and 10 did not consent to participate. The remaining 35 were randomized to the two arms of the study, 18 to intervention arm and 17 to the control arm.

Health care providers in these clinics consist of nurses, community health officers and community health extension workers. Each of these categories of providers has a minimum of two to three years of post-secondary education. Supervision for all the clinics in an LGA is provided by one general practitioner who is designated as the Primary Health Care Coordinator for the Local Government.

The study procedures were described to the supervising physicians in each of the LGAs and to the matrons or facility managers in each of the clinics. Only clinics providing explicit consent to participate were randomized into the trial.

### Randomization

Eligible and consenting primary health care clinics were stratified by local government area and allocated to intervention or control arm using a computer-generated random number sequence. Allocation was conducted by one of the authors (AAM) using anonymous codes for clinics and LGAs provided by other members of the research team, in order to avoid any risk of selection bias.

### Ethics and research governance

General information about depression is provided to all consecutive attendees in the waiting area of the clinic by trained research staff, after which their permission is sought for the screening interview. Those who screen positive (PHQ-9 score of 11 and above) have the full study protocol explained to them and willing participants sign the consent form.

Full ethical approval to conduct STEPCARE was obtained from the University of Ibadan/University College Hospital Joint Ethics Committee.

Adherence to Committee’s specifications and approved protocol is monitored by two independent groups: a Data Monitoring and Ethics Committee (DMEC) and a Trial Steering Committee (TSC). Both the DMEC and TSC conduct regular reviews of the field work, have one annual scheduled face-to-face meeting and twice yearly teleconferences. The role of the DMEC is to advise the TSC on issues relating to safety and ethical conduct of the study while the TSC has the responsibility of providing overall oversight for the study, including ensuring its implementation according to the approved protocol. The day-to-day implementation of the study is co-ordinated by the Study Management Team whose members include all the investigators, a Trial Manager and supervisors. The team meets bi-monthly by teleconference and annually face-to-face.

### Recruitment of participants and eligibility

Consecutive attendees at the selected PHCCs are approached while waiting to see the health care providers and are screened for depression using the 9-item patient health questionnaire (PHQ-9). Those who screen positive (PHQ-9 score 11 and above) are assessed for eligibility and invited to take part in the study. All consenting adults patients, aged 18 years and above with a score of 11 or more on PHQ-9 with confirmed DSM-IV diagnosis of major depression (depression diagnosis is confirmed using the short form of the Composite International Diagnostic Interview (CIDI) [16, 17] are eligible to participate. Patients are ineligible if there is an immediate need for medical attention, they are pregnant or nursing mothers, are actively suicidal, have a history of bipolar or psychotic disorder or of severe substance dependence. Also ineligible are those who are unlikely to remain in the neighbourhood over the following 12 months.

### Treatment in the intervention arm

Eligible and consenting patients are handed their PHQ-9 score and directed to see one of the trained PHCWs in the clinic. The PHCW takes further history to establish duration of symptoms, presence of any emergency (medical emergency or suicidality) and determines what intervention to administer.

The intervention incorporates components of the WHO mhGAP-Intervention Guide (MHGAP-IG) for depression, contextualized and adapted for the Nigerian health system [18, 19], and Problem Solving Treatment (PST) as used successfully in other interventions in LMIC [20] and in our pilot study [21]. The MHGAP-IG is designed to facilitate the recognition and management of a set of priority mental, neurological, and substance use (MNS) disorders in non-specialist settings. The depression module describes approaches for the recognition as well as the pharmacological and non-pharmacological treatment of depression. Problem solving approaches have proven to be successful in the treatment of common mental disorders such as depression and anxiety [20].

**Fig. 1:**
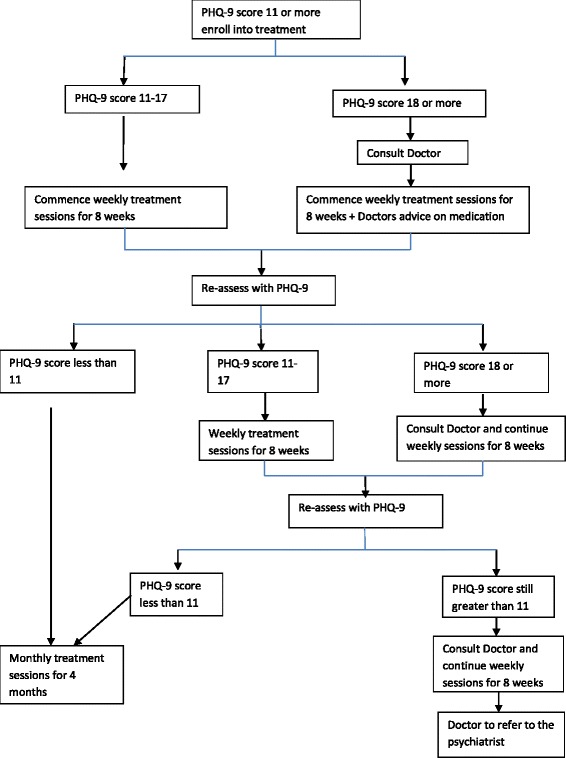
Treatment flow chart

The intervention is pragmatic, based on a stepped-care model, and is fully manualized. It is designed to be delivered in three steps determined by the patient’s score on the PHQ-9 and response to treatment (See Fig. 1). All interventions are carried out in the Yoruba language by health care providers fluent in the language and experienced in practicing in the locality. The interventions have been adapted to the local context and tested during a pilot study [21].

All individuals consenting to the trial receive **step 1.** In step 1, participants with PHQ-9 score between 11 and 14 receive only psychological interventions delivered by the PHCWs while those with PHQ-9 ≥ 15 at baseline are immediately assessed with the aim of initiating antidepressant medication in addition to the psychological treatment. Antidepressant medication is initiated following a discussion of the results of the assessment of the patient by the PHCW with the supervising general physician, using mobile phone. After 8 weekly sessions, all participants are re-assessed with the PHQ-9 and those who have not shown much improvement or whose symptoms worsen (PHQ-9 > 50 % of baseline score or ≥11) are moved to **step 2.** Participants in step 2 who have not previously been on antidepressant medication are reviewed in consultation with the GP with a view to initiating antidepressant medication and those who are already on medication are similarly assessed by the GP with a view to modifying medication regime. All participants who do not improve after this step may have their cases discussed with a psychiatrist by the GP in the final **step 3** in the sequence covering up to 6 months. The manual provides full description of each step and the required clinical decisions. At each visit, the PHCW asks structured questions to identify participants at risk of suicide, or who develop adverse reactions to medication. Such participants are flagged as an emergency and the GP is contacted immediately for consultation. All supervision and consultations with doctors are provided by mobile phones except when a face-to-face review is deemed necessary and feasible.

The psychological component of the intervention consists of psychoeducation, reactivation of social network, and PST. This intervention is delivered in 8 weekly sessions to all participants entering the program regardless of the need for medication. All sessions are carried out face-to-face in the clinic. Each session lasts approximately 30-45 min and are scheduled at times agreeable to both the patient and the PHCW. The initial session is dedicated to psychoeducation in which the symptoms of depression, possible causes and treatments are discussed. The following 5 sessions are focused on the basics of PST by working with the patient to identify and explore solutions to difficulties/problems they are currently facing. The PST in Session 6 is specifically dedicated to exploring support through social networks and the last two sessions are about integrating it all and preparing for the future.

The first line medication is amitriptyline, which non-physician primary care providers in Nigeria are authorized to prescribe. Other antidepressants could be prescribed by the GPs for patients who do not improve or have other contraindications to the use of tricyclic antidepressants. PHCWs are expected to consult with the GP when PHQ-9 denotes severe depression (PHQ score of 15 and above), there is no improvement at week 8 or in case of emergencies (e.g. suicidality or serious drug reaction).

#### Control arm

Participants in the control clinic receive ‘enhanced usual care’. Usual care is ‘enhanced’ by the training of the providers in this arm before the commencement of the trial on the recognition and management of depression. Subjects who are recruited in the control clinics are informed of their PHQ-9 scores and advised to show these to their health care providers. The choice of treatment offered is left to the discretion of the PHCW and consist of the usual services normally available in the clinics; these include antidepressant medications, brief psychotherapeutic interventions, medical consultations, or referral for specialty treatment. Although all these options are potentially part of usual care, in reality, unstructured counselling is often all a patient with recognized depression receives.

### Training

Prior to recruitment of patients, providers in the intervention arm received training on the recognition of depression, the delivery of the manualized intervention package, how to obtain and document support and supervision received from the GP using mobile phones. The training consisted of didactic lectures, clinical demonstrations and role plays over a 3-day period. They had a further 2-day top-up training about a month into the study to reinforce the acquired skills and review experience with implementation of the intervention.

Training for the providers in the control arm was conducted separately. They received a 2-day training on the identification and treatment of depression. This training was based on the mhGAP-IG but without detailed PST training or guidelines and procedure for obtaining structured support and supervision from physicians. That is, the providers in the control arm were trained in the recognition and standard treatment of depression, but not in the use of PST or the implementation of a stepped-care management approach (see below).

### Support and supervision in the stepped-care model

The components and tasks for each treatment session as well as the clinical decisions and steps are detailed in manuals and charts provided to the PHCW and primary care physicians. Mobile telephone lines were provided to each of the trained PHCW in the intervention clinics and their supervising GPs and study psychiatrist. These mobile phone lines are linked in a closed user group network where calls within the network are free to facilitate consultation. All telephone reviews and consultations are on as-needed basis, structured, and follow a flow-chart that proceeds from the PHCWs through to the GP and to the psychiatrist.

### Outcome measures

#### Primary

The primary outcome is the proportion of patients who recover from depression at 12 months from entry to the trial. Recovery from depression is defined as a PHQ-9 score < 6.

#### Secondary

Secondary outcomes are assessed at 6 and 12 months and consist of: 1) change in depression symptoms at 6 months; 2) level of disability as assessed using the WHO Disability Assessment Scale; [22] 3) Quality of life as measured using the WHO Quality of Life instrument [23] and 4) health care utilization, assessed with the Service Utilization Questionnaire (SUQ), adapted for the purpose. The SUQ is derived from the Client Service Receipt Inventory (CSRI) which is designed to collect information about the use and costs of health and social services and other economic impacts such as time of work due to illness [24, 25]. The unit costs or prices of these various resource inputs will be based on the results of a costing analysis which we have conducted in a number of health facilities in the setting of the trial.

We have used the scales included in this protocol in our previous studies as well as during our pilot study and have found them to be acceptable to patients and sensitive to change [21, 26, 27]. All outcome assessments are administered in face-to-face interviews at the respondents’ homes by trained interviewers using the Yoruba versions of the different instruments. The Yoruba versions were derived by standard protocols of iterative back translations and have been used in previous surveys with good psychometric properties. Outcome assessors are not involved in delivering the intervention and are rotated between PHCCs to collect data. We will seek to collect outcome data from every participant not known to have died at the time of follow-up and who has not withdrawn consent, regardless of compliance with allocated treatment.

#### Economic evaluation

We plan to carry out an economic evaluation. Using the SUQ, we will systematically collect resource-use data, including any inpatient care, consultations with health providers, use of drugs and laboratory tests, and also time and travel costs associated with this service uptake. We will also collect information on the financing sources for each of the categories in order to allow for an estimation of the extent of private, out-of-pocket expenditures incurred by study subjects and their families. The unit costs or prices of these various resource inputs will be derived by carrying out costing analysis in a number of participating health facilities using data collection templates and protocols previously developed and applied by us.

Since depression and associated disability outcomes for the stepped care intervention are also expected to improve significantly, the intervention will 'dominate' usual care (i.e. better outcomes, less cost). Such a hypothesis negates the need for a power calculation. If, however, costs turn out to be higher in the intervention group, bootstrapped incremental cost-effectiveness ratios for PHQ-9 depression and WHO-DAS disability scores level will be derived. Using the results of the Nigerian sample of the WHO multi-country survey study on health and health system responsiveness to convert WHO-DAS summary score to a health state preference measure, we will also construct Quality Adjusted Life Years (QALYs) for both groups, thereby allowing comparison of this intervention with other evaluations undertaken in Nigeria and elsewhere. Whether point estimates demonstrate dominance or not, results will be plotted on a cost-effectiveness plane and presented as cost-effectiveness acceptability curves in order to show the probability of the intervention being cost-effective at a range of 'willingness-to-pay' threshold levels. We will conduct sensitivity analysis to take account of uncertainty and imprecision in the measurements, including multiple imputation models for missing values.

### Sample size and power calculation

Previous studies have shown that low to moderate intensity treatment for depression yields effect sizes on a variety of questionnaire-based outcomes of about 0.33 standard deviations, and about 50 % relative advantage in recovery rate compared to usual care [28]. Experience from our previous PHC studies as well as from the control arm of the Chilean trial suggest a recovery rate of about 30 % for major depression with no active treatment and about 70 % with treatment [29]. For our sample size estimation, we sought to detect an absolute difference of 18 percentage points (41 % recovery in control and 59 % in intervention groups respectively) at 12 months, a difference that we think is both plausible for this type of intervention and would promote changes in practice. We assumed an intra-cluster correlation coefficient (ICC) of 0.05 based on pilot study data, and collection of the primary outcome for 80 % of participants. The uninflated sample size requires 131 per arm for analysis to detect a difference of 59 % vs 41 % (equivalent odds ratio = 2.1) with 80 % power at the two-sided 5 % alpha level. We aimed to recruit 90 individuals per clinic. With 72 per cluster available for the primary analysis and an ICC of 0.05, the design effect is 4.55, giving a total number required for analysis of 1190. We therefore aimed to recruit 90 individuals from each of 16 clinics initially. As participant recruitment was slower than anticipated, in March and November, 2014 we recruited and randomised a further 19 clinics, giving a total of 35 in the study. Participant recruitment started December 2013 and is still ongoing.

### Data analysis

Individual data is collected and stored electronically using palmtops programmed to capture information directly from respondents. This is to ensure accuracy and security of data collection. All data are kept anonymously using codes to identify individuals. Data is downloaded from palmtops to desktops located in the central office in Ibadan where it will be cleaned and stored. These datasets do not contain the allocation status of the participants which is kept as a separate file and only for the trial statistician. Access to the datasets is possible for members of the research team through a password-protected entry.

A full statistical analysis plan will be developed before any data are analyzed. The analysis and presentation of the trial will be in accordance with CONSORT guidelines for cluster randomized trials [30, 31]. The primary approach for comparative analyses will be to analyze participants as randomized without imputation of missing data, and with due emphasis placed on confidence intervals for the between-arm comparisons. We will use descriptive statistics to assess balance between the trial arms at baseline for both clinic and individual participant characteristics. In order to take appropriate account of the hierarchical nature of the data, we will use multivariable mixed effects regression models to estimate recovery from depression at 12 months for intervention group versus control, adjusting for baseline depression and LGA as a stratification variable. In a secondary analysis, we will further adjust for any variables that were imbalanced between trial arms at baseline. These analyses will be repeated for secondary outcomes. We will conduct sensitivity analyses to assess the potential effect of missing data, and will investigate the effect of adherence with the intervention. We will investigate whether between-group differences vary over time using data from all follow-up visits in repeated measures analyses.

We will investigate whether there is any differential effect of the intervention according to baseline symptom severity (PHQ-9 score <16, ≥16) and duration (≤3 months, >3 months) by including appropriate interaction terms in the primary regression model. Since the trial is powered to detect overall differences between groups rather than interactions of this kind, the results will be interpreted with due caution.

Data analysis will be conducted once all follow-up is complete. There are no planned interim analyses.

## Discussion

This randomized-controlled trial evaluates the effectiveness of a stepped care intervention model for depression in primary care compared to enhanced care as usual. The key strength of this study is the use of a task-sharing model that relies almost entirely on the capability of non-physicians PHCWs to deliver an evidence-based intervention, in keeping with the scarcity of specialist mental health manpower in the country. The stepped care model examines the effectiveness of an intervention package for depression in which the intensity of treatment is determined by the clinical need of the patients.
